# Prognostic Role of Ventricular Pacing Burden in Patients with Pacemaker Implantation After TAVR

**DOI:** 10.3390/medicina61101758

**Published:** 2025-09-28

**Authors:** Abdullah Orhan Demirtas, Abdullah Yildirim, Mukremin Coskun, Hasan Burak Ozdemir, Emre Sezici, Ibrahim Halil Kurt

**Affiliations:** Department of Cardiology, University of Health Sciences, Adana City Training and Research Hospital, 01230 Adana, Türkiye; dr.yildirimabdullah@gmail.com (A.Y.); mukremincoskun@gmail.com (M.C.); hsn.burakozdemir@gmail.com (H.B.O.); emresezici@gmail.com (E.S.); ibrahimhalilkurt@gmail.com (I.H.K.)

**Keywords:** transcatheter aortic valve replacement, TAVR, permanent pacemaker, ventricular pacing, conduction disturbances, atrioventricular block

## Abstract

*Background and Objectives:* Permanent pacemaker (PPM) implantation after transcatheter aortic valve replacement (TAVR) is common, yet the prognostic implications of ventricular pacing (VP) burden remain uncertain. *Materials and Methods:* We retrospective single-center cohort study included 118 patients undergoing TAVR who required new PPM implantation. Using X-tile analysis, patients were stratified into two groups, VP < 85% and VP ≥ 85%, at 1 month. Baseline characteristics, post-procedural outcomes, echocardiographic parameters, and long-term endpoints were compared. The primary endpoint was all-cause mortality; the secondary endpoint was a composite of mortality and first heart failure (HF) hospitalization. *Results:* At 1 month, 73 patients (61.9%) had VP < 85% and 45 (38.1%) had VP ≥ 85%. Baseline demographics were similar, but patients with VP ≥ 85% had higher LVEF (59.4 ± 4.2 vs. 49.4 ± 6.7%, *p* < 0.001), lower creatinine (0.8 vs. 1.0 mg/dL, *p* = 0.016), and higher e-GFR (69.6 ± 20.6 vs. 60.4 ± 19.8, *p* = 0.017). Time to PPM implantation was shorter in the VP ≥ 85% group (2.6 ± 1.3 vs. 6.2 ± 2.6 days, *p* < 0.001). During a median follow-up of 315 ± 105 days, mortality did not differ between groups (HR: 1.81, 95% CI: 0.76–4.31, *p* = 0.182). However, VP ≥ 85% was associated with a higher incidence of the secondary endpoint (HR: 2.36, 95% CI: 1.21–4.58, *p* = 0.012). Echocardiographic follow-up revealed lower LVEF and higher e-SPAP at 1 year in the VP ≥ 85% group (both *p* < 0.001). *Conclusions:* In TAVR patients requiring PPM, a high VP burden (≥85%) was not associated with increased mortality but predicted a higher risk of HF hospitalization and deterioration in echocardiographic parameters.

## 1. Introduction

Aortic stenosis is a common valvular disease in the elderly and is associated with high mortality once symptoms develop. In recent years, transcatheter aortic valve replacement (TAVR) has emerged as a safe and effective treatment alternative to surgical aortic valve replacement (sAVR) owing to its lower mortality and complication rates [1,2]. However, the procedure remains associated with several significant complications. One of the leading complications is atrioventricular block (AVB) and other conduction disturbances that develop after TAVR due to the close anatomical proximity of the aortic annulus to the conduction system [3]. These conduction disturbances, especially complete AVB, may necessitate permanent pacemaker (PPM) implantation. Reported PPM implantation rates range from 2% to 36%, depending on valve type, generation of devices, patient risk categories, and implantation techniques [4,5,6,7]. The need for PPM after TAVR has been associated with electrocardiographic findings such as right bundle branch block (RBBB), new-onset left bundle branch block (LBBB), or prolonged PR/QRS intervals after the procedure. However, in clinical practice, decisions are often influenced by physician experience, extending beyond the indications clearly defined in the European Society of Cardiology (ESC) guidelines, which contributes to heterogeneity.

A considerable proportion of patients undergoing PPM implantation due to post-TAVR conduction disturbances experience very low ventricular pacing (VP) rates, raising the question of the need for PPM. Conversely, studies have demonstrated that high right VP rates resulting from high-degree AVB requiring PPM implantation may attenuate the long-term benefits of TAVR, promote cardiac dysfunction and heart failure (HF), and contribute to adverse outcomes [8]. Continuous high-burden VP, which induces ventricular asynchrony, can precipitate HF, particularly in patients with pre-existing left ventricular (LV) dysfunction.

In this context, our study aimed to evaluate the relationship between VP rates and one-year mortality in patients who underwent PPM implantation due to the development of AVB after TAVR. We also aimed to examine the relationship between VP percentage and HF findings and draw prognostic implications for clinical practice.

## 2. Materials and Methods

### 2.1. Study Design and Setting

This retrospective single-center cohort study included patients who underwent TAVR between September 2017 and June 2024. The study population consisted of patients who received a dual-lead (DDD mode) or single-lead (VVI mode) PPM due to early postprocedural high-grade AVB or intermittent conduction disturbances. Patients were excluded if they underwent biventricular pacing procedures or left bundle branch area pacing (LBBAP) (n = 4), had PPM implantation before TAVR due to AVB (n = 6), or lacked follow-up data (n = 4). A total of 115 patients with newly implanted PPMs and 3 patients who had a pre-TAVR dual lead ICD but developed AVB after the procedure (despite having no prior conduction abnormalities) were included. The final analysis included 118 patients.

### 2.2. TAVR Procedure and Permanent Pacemaker Implantation

Decisions regarding TAVR procedures were made by a heart team, considering local clinical practices, national healthcare regulations, and current international treatment guidelines. Demographic data, medical history, and patient characteristics were obtained from hospital and national health records and documented by an independent local heart team. The study utilized balloon-expandable transcatheter heart valves (THVs) such as Myval (Meril Life Sciences, Vapi, India), and self-expandable THVs including Portico-Navitor (Abbott Structural Heart, Minneapolis, MN, USA), Evolut R (Medtronic, Minneapolis, MN, USA). The preferred access route for all cases was percutaneous transfemoral access; if unsuitable (due to tortuosity, inappropriate diameters, severe calcification, or extensive iliofemoral atherosclerosis), subclavian/axillary access was chosen. For all included patients, multidetector computed tomography was used to determine aortic measurements, valve size selection, valve calcification, and the suitability of iliofemoral arteries. The choice of valve size and type, the need for pre- or post-dilation, use of rapid pacing, and type of anesthesia were left to the discretion of the operational team. The implantation procedure was conducted in a catheterization laboratory with adequate equipment and under angiographic guidance. For vascular closure, the Perclose ProGlide (Abbott Structural Heart, Minneapolis, MN, USA) device was used in all patients.

The decision for PPM implantation was made by two independent electrophysiologists and one interventional cardiologist, in line with current guidelines. High-grade AVB (third-degree or Mobitz type II second-degree AVB) was considered an absolute indication for postprocedural PPM implantation. In cases without definitive indication (e.g., bifascicular block, bifascicular block with PR prolongation, severe bradycardia), 24 h Holter monitoring and electrophysiological studies were performed as needed. The decision between single- or dual-chamber PPM implantation was made according to the patient’s clinical condition by the same team who recommended PPM implantation. Antibiotic prophylaxis and venography were performed before the procedure, and PPM implantation was conducted via the axillary approach. The ventricular lead was positioned in the right ventricular (RV) low septum without discontinuing antiplatelet therapy. In patients receiving an atrial lead, the lead was positioned in the right atrial appendage.

Echocardiographic examinations were conducted by an independent and experienced cardiologist at the imaging center of the clinic using the ACUSON SC2000 Prime (Siemens Medical Solutions, Mountain View, CA, USA) echocardiography device before implantation, as well as at 1 month after. According to the most recent recommendations, the heart’s chambers and functions, valves, ejection fraction, and other echocardiographic data were assessed. Based on the continuity equation, AVA was calculated [9]. Left ventricular ejection fraction (LVEF) was calculated using the Simpson method, and estimated systolic pulmonary artery pressure (e-SPAP) was derived from tricuspid regurgitant flow according to the continuity equation [9].

The study was conducted in accordance with the Declaration of Helsinki and was approved by the local ethics committee (date: 7 November 2024; decision number: 7/224). Written informed consent was obtained from all participants.

### 2.3. Study Protocol and Definitions

The standardization of all TAVR-related outcomes in this study was defined according to the 2021 updated Valve Academic Research Consortium (VARC)-3 recommendations [3]. The primary endpoints of the study were all-cause mortality at 12 months. The secondary endpoint was the composite of all-cause mortality or first hospitalization for HF, stratified by VP percentage. 1-year follow-up data were obtained through hospital visits or telephone interviews. The VP percentage was calculated at the 1-month follow-up for all patients. New York Heart Association (NYHA) functional class was assessed at pre-TAVR, at 12 months, and at last available follow-up.

### 2.4. Statistical Interpretation

Data and statistical analyses were conducted using X-tile software, v3.6.1 (Yale University, New Haven, CT, USA), and R software v4.5.0 (Vienna, Austria). Categorical data were reported as counts and proportions, while continuous data were expressed as mean ± standard deviation (SD) or median (interquartile range, IQR: 25th–75th percentile), depending on the distribution pattern. The Shapiro–Wilk test and visual inspection of histograms were used to assess the normality of continuous variables. Between-group differences were evaluated using the chi-square test or Fisher’s exact test for categorical variables, and Student’s *t*-test or Mann–Whitney U test for continuous variables, as appropriate. Event-free survival was estimated using Kaplan–Meier curves, and group comparisons were performed using the log-rank test. Proportional hazards assumptions for Cox regression models were verified using Schoenfeld residuals and inspection of log–log plots. Results were presented as hazard ratios (HRs) with 95% confidence intervals (CIs). The VP percentage was analyzed both as a categorical and continuous variable.

The optimal cut-off point for VP percentage was determined using the X-tile which identified 85% as the threshold. To explore non-linear associations, VP was also modelled as a continuous exposure using restricted cubic splines with four knots (5th, 35th, 65th, 95th percentiles). For echocardiographic outcomes group differences were analyzed using ANCOVA. NYHA functional capacity was compared across VP groups at baseline, 12 months, and last follow-up using chi-square tests and the linear-by-linear association test. The discriminative ability of VP percentage for clinical outcomes was evaluated using time-dependent ROC analysis at 90, 180, 270, and 360 days, with Uno’s estimator and IPCW (inverse-probability-of-censoring weighting). All tests were two-tailed, and a *p*-value < 0.05 was considered statistically significant.

## 3. Results

### 3.1. Baseline Characteristics, Procedural Data, and Outcomes

**Figure 1:**
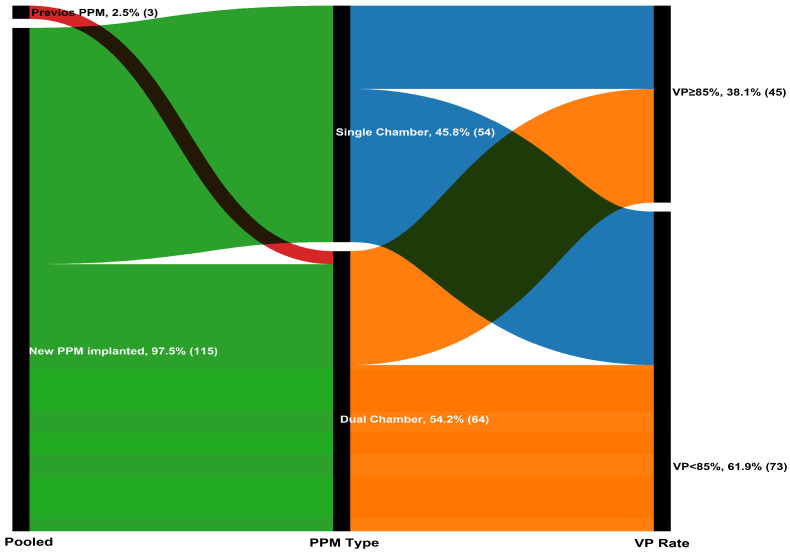
The Sankey diagram illustrating the progression of 118 patients through the pacing stages.

A total of 118 patients (mean age: 77.9 ± 6.7 years; 50.8% women) were included in the analysis. Based on the optimal cutoff determined by X-tile analysis (maximum chi-square value), patients were stratified into two groups: VP < 85% and VP ≥ 85% (Supplementary Figure S1). Of these, 73 patients (61.9%) had VP < 85% and 45 (38.1%) had VP ≥ 85% at 1-month follow-up (Figure 1). Demographic characteristics were comparable between the two groups (all *p*-values > 0.05). Regarding laboratory parameters, patients with VP ≥ 85% had lower baseline creatinine (1.0 vs. 0.8 mg/dL, *p* = 0.016) and higher e-GFR (60.4 ± 19.8 vs. 69.6 ± 20.6, *p* = 0.017), higher NT-proBNP (760 vs. 1682 pg/mL, *p* = 0.036), higher baseline LVEF (49.4 ± 6.7 vs. 59.4 ± 4.2%, *p* < 0.001), and lower e-SPAP (34 vs. 32 mmHg, *p* = 0.045). Baseline demographic characteristics were balanced between the two groups (all *p* > 0.05) (Table 1)

### 3.2. Post-TAVR Outcomes

Procedural features such as valve type, pre-dilatation, and post-dilatation rates were comparable between the groups. Time to PPM implantation was shorter in patients with VP ≥ 85% (6.2 ± 2.6 vs. 2.6 ± 1.3 days, *p* < 0.001). The predominant indication for PPM implantation in patients with VP ≥ 85% was complete high-degree AVB (71.1% of patients), while paroxysmal high-degree AVB was the most frequent indication in patients with VP < 85% (31.5% of patients, *p* < 0.001). The type of PPM implanted and complication rates were similar across groups. On post-TAVR electrocardiography, patients with VP < 85% showed a similar incidence of conduction disorders (RBBB with left anterior/posterior fascicular block: 13.7 vs. 4.4%, *p* = 0.106), but they more frequently developed new-onset atrial fibrillation (AF) (9.0 vs. 0.0%, *p* = 0.014) (Table 2).

**Figure 2:**
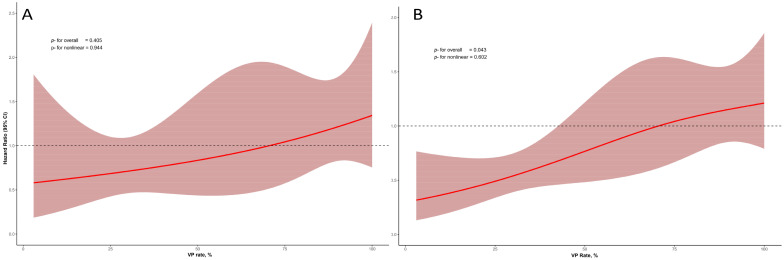
Restricted cubic spline curves illustrating the association between VP rate and clinical outcomes. (**A**) Relationship between VP rate and all-cause mortality. (**B**) Relationship between VP rate and the composite outcome of all-cause mortality and first HF hospitalization.

Figure 2 shows the non-linear relationship between VP rate and outcomes, which was particularly evident when VP was ≥85%. In restricted cubic spline analysis, no significant overall association was observed between VP rate and mortality (*p* = 0.405), and there was no evidence of nonlinearity (*p* = 0.944). In contrast, for the composite endpoint of mortality and HF hospitalization, a significant overall association was observed (*p* = 0.043), while the nonlinear component was not significant (*p* = 0.602).

**Figure 3:**
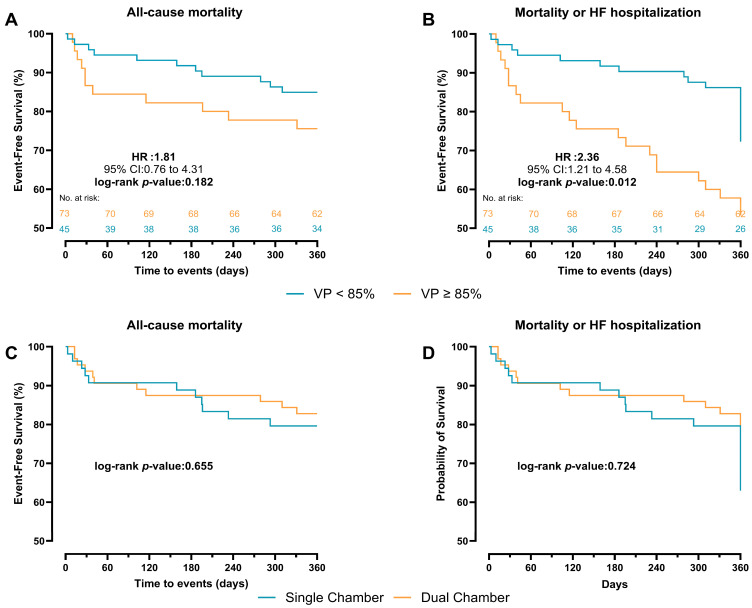
Kaplan–Meier survival curves comparing VP < 85% and ≥85% pacing for 1-year outcome. (**A**) all-cause mortality. (**B**) first HF hospitalization or mortality. Kaplan–Meier survival curves comparing chamber type for 360-day outcome. (**C**) all-cause mortality. (**D**) first HF hospitalization or mortality.

During a mean follow-up of 315 ± 105 days, the primary endpoint occurred in 11 patients (15.1%) with VP < 85% and 11 (24.4%) with VP ≥ 85%. The VP rate was higher in deceased patients compared with survivors (70.5 ± 33.1 vs. 59.5 ± 35.0). In Cox regression analysis, VP burden as a continuous variable was not significantly associated with all-cause mortality (a-HR: 1.02, 95% CI: 0.99–1.04, *p* = 0.077). Categorically, VP ≥ 85% was associated with a higher, though non-significant, risk of mortality compared with VP < 85% (a-HR: 2.04, 95% CI: 0.61–6.78, *p* = 0.244) (Table 3). In Kaplan–Meier event-free curves, patients with VP ≥ 85% had a similar cumulative incidence of the primary endpoint compared with patients with VP < 85% (HR: 1.81, 95% CI:0.76–4.31, log-rank *p* = 0.182) (Figure 3A). However, patients with VP ≥ 85% had a higher cumulative incidence of the secondary endpoint (first HF hospitalization and mortality) compared with those with VP < 85% (HR: 2.36, 95% CI: 1.21–4.58, log-rank *p* = 0.012) (Figure 3B). In the unadjusted Kaplan–Meier curves, patients with single-chamber PPM implanted showed a similar incidence of both the primary and secondary endpoints compared with VP ≥ 85% (log-rank *p* = 0.655 and *p* = 0.724, respectively) (Figure 3C,D).

**Figure 4:**
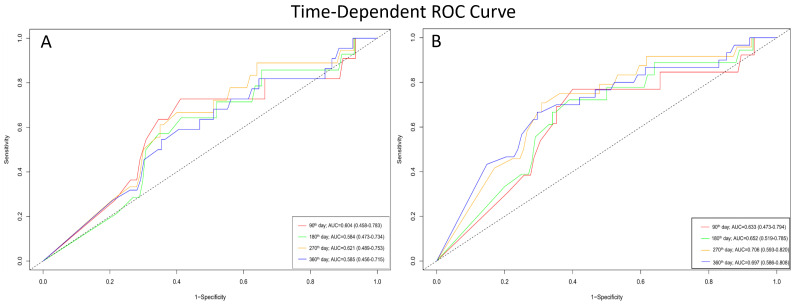
Time-dependent ROC curves demonstrating the prognostic performance of VP rate at different follow-up times. (**A**) Accuracy of VP rate in predicting all-cause mortality. (**B**) Accuracy of VP rate in predicting the composite outcome of all-cause mortality and first HF hospitalization.

In patients with VP ≥ 85%, time-dependent ROC analysis for all-cause mortality yielded AUC values of 0.604 (95% CI: 0.458–0.783) at 90 days, 0.584 (95% CI: 0.473–0.734) at 180 days, 0.621 (95% CI: 0.489–0.753) at 270 days, and 0.585 (95% CI: 0.456–0.715) at 360 days (Figure 4A). For the secondary outcome of mortality and HF-related hospitalization, the AUC values were 0.633 (95% CI: 0.473–0.794) at 90 days, 0.652 (95% CI: 0.519–0.785) at 180 days, 0.706 (95% CI: 0.593–0.820) at 270 days, and 0.697 (95% CI: 0.586–0.808) at 360 days (Figure 4B).

### 3.3. Echocardiographic Features and Functional Status

**Figure 5:**
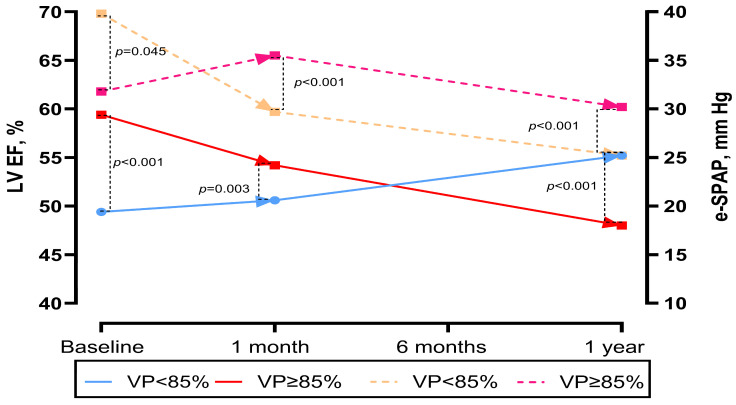
Changes in left ventricular ejection fraction and e-SPAP over time in the pooled population and in patients according to VP percentage.

LVEF at baseline, 1 month, and 1 year post-TAVR was available for 92 patients (77.9%) alive at 1 year. At 1 month, patients with VP ≥ 85% had lower LVEF (50.6 ± 6.6 vs. 54.2 ± 5.8%, *p* = 0.003). Over time, patients with VP ≥ 85% demonstrated a less favourable trend in LVEF change (control LVEF—baseline LVEF). At 1 year, patients with VP ≥ 85% continued to have significantly lower LVEF compared with those with VP < 85% (*p* < 0.001). A similar pattern was observed for e-SPAP: at baseline, e-SPAP was lower in the VP ≥ 85% group (34 vs. 32 mmHg, *p* = 0.045), whereas at 1 year, e-SPAP was higher in patients with VP ≥ 85% compared with those with VP < 85% (*p* < 0.001) (Table 2 and Figure 5).

**Figure 6:**
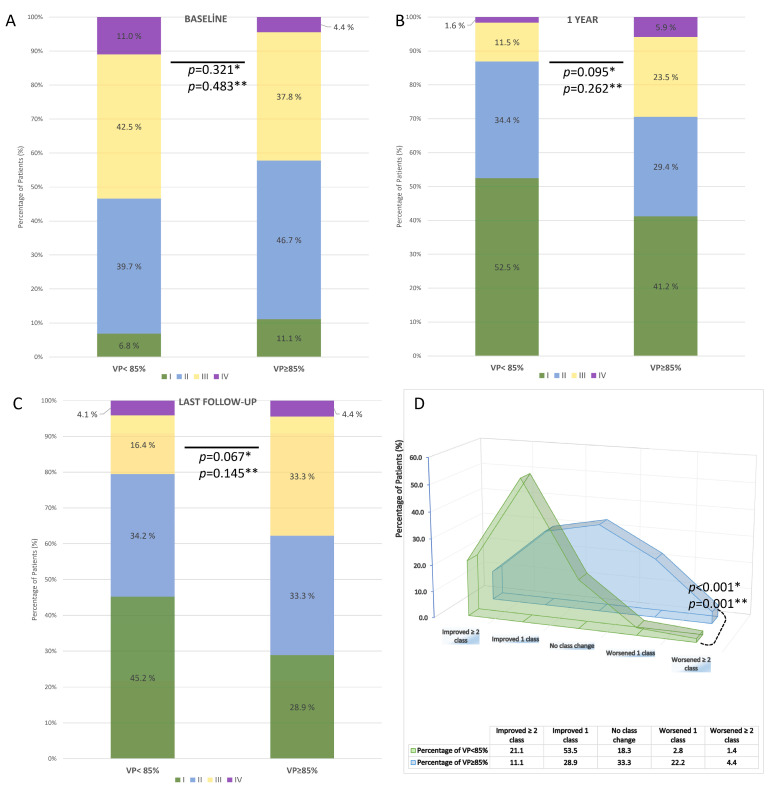
Distribution and change in NYHA functional class according to ventricular pacing (VP) rate after TAVR in patients with permanent pacemaker implantation. (**A**) Baseline distribution of NYHA class in VP < 85% vs. VP ≥ 85% groups. (**B**) NYHA class at 1-year follow-up. (**C**) NYHA class at the last available follow-up. (**D**) Change in NYHA functional class during follow-up. * A *p*-values are based on 2 × 2 chi-square tests; ** A *p*-values are based on 4 × 2 chi-square tests.

NYHA functional class data were available for 95 patients (80.5%) at 1 year and 116 patients (98.3%) at the last available follow-up. Compared with baseline, both groups demonstrated overall improvement in NYHA class. At baseline, there was no significant difference between VP < 85% and VP ≥ 85% groups (*p* = 0.321) (Figure 6A). At 1 year, functional class remained similar between the groups (*p* = 0.095) (Figure 6B), whereas at the last follow-up, patients with VP ≥ 85% showed a trend toward worse NYHA distribution compared with those with VP < 85% (*p* = 0.067 for 2 × 2; *p* = 0.145 for 4 × 2) (Figure 6C). Analysis of NYHA change revealed that 74.6% of patients with VP < 85% improved by ≥1 class, compared with only 40.0% of patients with VP ≥ 85%. Conversely, no improvement or worsening was observed in 60.0% of patients with VP ≥ 85% vs. 25.4% with VP < 85%. This difference was statistically significant (*p* < 0.001) (Figure 6D).

## 4. Discussion

The main findings of our study investigating the importance of VP ratio in follow-up in patients undergoing early PPM after TAVR are as follows: (i) While the VP ratio in patients undergoing PPM implantation after TAVR is related to the reason for implantation, it is not related to factors such as age, valve type, LV function, or AF. (ii) In patients with VP ≥ 85%, improvement in NYHA functional class after TAVR was less, while some patients experienced worsening. (iii) Mortality and HF-related hospitalizations were more common in patients with VP ≥ 85% during the 1-year follow-up after TAVR.

Conduction disturbances and arrhythmias such as newly developed AF, LBBB, and high-grade AVB are more common after TAVR than after surgical aortic valve replacement. PPM implantation, particularly with first-generation THV around 35–54%, is not considered an endpoint in the current VARC-3 criteria [3]. Nevertheless, the outcomes of pacemaker and/or implantable cardioverter-defibrillator implantation after TAVR need to be better understood [8,10]. Research on this topic should focus specifically on the use of pacing during TAVR, post-TAVR new PPM implantation rates, and developments that will reduce pacing requirements [4,5,8,11]. However, the prognostic impact of PPM after TAVR and its long-term outcomes remain controversial [8,11,12]. Moreover, studies on TAVR and post-procedural PPM implantation provide conflicting results regarding mortality and hospitalizations due to HF. Jørgensen et al. reported that PPM implantation was performed in 16.2% of patients after TAVR and that there was no difference in 1-year mortality compared to those without conduction abnormalities [13]. Yıldırım et al. reported a new PPM rate of 12.2% after TAVR and similarly reported no difference in mortality [6]. However, these studies have short follow-up periods and are generally focused on mortality or cardiac mortality outcomes. However, with increasing TAVR cases, expanding indications, widespread use of TAVR in low-risk populations, and a younger patient population, outcomes such as functional recovery and recurrent hospitalizations, in addition to mortality, have become important. A recent comprehensive meta-analysis reported an increase in HF hospitalizations and mortality in patients implanted with PPM after TAVR. Furthermore, this difference persisted with long-term follow-up [14]. Another registry reporting a mean follow-up of 3420 TAVR patients with an average of 2.7 years reported no difference in cardiovascular mortality or HF with regard to PPM [12].

In the literature, studies of patients with PPM implantation after TAVR provide limited information on the effect of right VP percentages on different outcomes. Hochstadt et al. compared patients with high VP load to those with low VP load and reported similar long-term mortality, while decreased LVEF was more common [15]. Nadeem et al. found no difference in mortality in 30 patients with VP ≥ 40% at one-year follow-up but reported significantly higher rates of hospitalization for HF [16]. Bruno et al. found similar mortality rates in TAVR patients with PPM implantation with VP < 40% and ≥40% but reported that hospitalizations for HF and overall clinical outcomes were less favourable in patients with VP ≥ 40%. Furthermore, LVEF recovery and change in NYHA class were also less favourable in the group with VP ≥ 40% [8]. These studies have highlighted the negative effects of right VP on long-term LV remodelling [8,15,16]. Similarly, in our study, no significant difference in mortality was observed in patients with high pacing loads, while the incidence of HF hospitalization was significantly higher. Additionally, although we did not find any difference between the groups in NYHA class, NYHA change, LVEF, and e-SPAP levels were worse in these patients at follow-up. The conclusion drawn from this is that the VP rate should be considered not only as an electrophysiological parameter but also as a prognostic marker in each patient.

High right VP rate can lead to a non-physiological electrical activation pattern, leading to both intra- and interventricular dyssynchrony, ultimately leading to long-term decreased LVEF and deterioration in NYHA functional capacity [8,16,17]. In the MOST study, a VP of >80% increased the risk of HF hospitalization by 2.5-fold, and every 10% increase in right VP increased the risk of HF hospitalization by 20% [17]. This suggests that the high rate of chronic right VP found in both previous studies and our study may be the reason behind the adverse outcomes. Furthermore, the heterogeneity of results between studies may be due to changes in the VP ratio. The fact that adverse right VP outcomes in PPM TAVR patients generally occur with short follow-ups of 1-year suggests that this population is more sensitive to non-physiological pacing due to overt or subclinical LV dysfunction, which is already common. In fact, it has been suggested that biventricular pacing and conduction system pacing should be considered in patients requiring permanent pacing and at risk of decreased LVEF due to a high VP ratio [18,19]. However, the use of biventricular pacing in TAVR patients has been either rarely reported or not reported in previous studies [8,16]. Hochstadt et al. implanted cardiac resynchronization therapy—defibrillator (CRT-D) only in patients with LVEF ≤ 35% and reported a rate of 2.2%. They excluded these patients from analyses based on VP load [15]. Nadeem et al. reported a rate of 0.0% for biventricular pacing [16]. In our study, we used CRT-D and LBBAP in only four patients, but we excluded these patients from the final analysis. Unlike RV pacing, high VP rates are essential for treatment response in these patients [18,19]. However, no mortality or hospitalization for HF was observed in the 1-year follow-up of these excluded patients. Although conduction system pacing in TAVR patients has been reported to yield more favourable outcomes compared to high right VP, most available studies are limited by small sample sizes and relatively short follow-up periods [20,21]. In contrast, Wang et al. demonstrated over a 5-year follow-up that LBBAP, compared with right VP, was associated with fewer HF-related hospitalizations and greater improvements in LVEF among TAVR patients requiring PPM implantation [22]. However, current guidelines provide specific recommendations for PPM implantation in post-TAVR patients; however, they do not include guidance regarding the preferred pacemaker types or pacing modes [19]. We used single- and dual-chamber pacemakers in our study. Complication rates were low, and unrelated to pacemaker type. Bruno et al., similarly, reported no difference between single and dual chamber pacemakers [8]. However, considering the risk of pacemaker syndrome during follow-up, long-term and well-designed studies are needed on this subject.

Our findings are largely consistent with previous reports in the literature, while providing additional insights in several aspects. First, we adopted a higher cut-off VP rate of 85% compared with other studies on TAVR and PPM [8,17]. We also reported that the most important factor determining the VP rate is the indication for PPM implantation. Similarly, Pelargonio et al. reported that the primary determinant of VP burden was the indication for PPM implantation. In addition, they suggested that in patients with new-onset conduction disturbances, such as BBB, individualized device programming and patient-specific strategies could reduce VP burden and potentially the need for PPM implantation [23]. In our study, although an increase in mortality was observed in the group with a higher percentage, this difference did not reach statistical significance. However, HF hospitalization rates and worsening functional parameters were observed more frequently in this group. Therefore, the indication for implantation and the potential need for VP rate should also be considered in patients undergoing PPM. Additionally, strategies such as physiological pacing approaches (e.g., LBBAP, his bundle pacing) should be considered in appropriate cases.

Our study did not reveal a direct relationship between PPM implantation after TAVR and early mortality. Furthermore, we confirmed that PPM implantation is safe and has a low complication rate, consistent with other studies and meta-analyses [24]. However, PPM implantation and prolonged high-burden VP are associated with late hospitalizations and worsening HF, thereby increasing patient complexity. Furthermore, increased hospital visits (pacemaker follow-ups and adjustments, generator replacement), as well as potential complications (lead-related endocarditis, lead dysfunction) associated with an additional cardiac device, should not be overlooked. Although modern pacing strategies are associated with a reduced risk of cardiomyopathy, their high cost, implantation difficulty, increased complication rates, and the fact that they have not yet become as widespread as TAVR pose serious problems [8,20,25]. Therefore, due to the nature of minimalist-TAVR, approaches such as CT-based anatomical concepts that reduce the need for PPM and avoid the RV [26], high implantation techniques such as cusp overlap [27], LV guidewire pacing without RV pacing [11,28], and THV technologies with low AVB rates should be supported and continue to be the focus of research [29,30].

This study has several limitations. First, the single-center and cross-sectional design limits the generalizability of our findings. Second, pacing burden was assessed using device interrogations, which may not fully capture real-time hemodynamic effects during patients’ daily activities. In addition, other pacing parameters such as AV delay optimization or device programming, which may also influence outcomes, were not systematically evaluated. Third, the study did not include comprehensive data on the use of contemporary guideline-directed medical therapy for HF (e.g., SGLT2 inhibitors, sacubitril/valsartan), and the absence of these data may have affected the interpretation of HF-related outcomes. Fourth, we assessed only the right VP burden without considering different pacing sites (e.g., His bundle pacing, LBBAP, or CRT), which are important factors influencing ventricular remodelling and clinical outcomes. Finally, the relatively small sample size may have reduced the statistical power of subgroup analyses. Larger, prospective multicenter studies are warranted, particularly for less frequent outcomes such as mortality.

## 5. Conclusions

This study demonstrated that a high-burden right ventricular pacing (≥85%) in patients undergoing PPM implantation after TAVR was associated with worse functional recovery and a higher rate of HF-related hospitalizations. Although no significant relationship with mortality was observed, improvement in NYHA class was more limited in patients with higher pacing rates, and some patients experienced worsening. These findings highlight the potential clinical importance of VP burden in the management of patients requiring PPM after TAVR.

## Figures and Tables

**Table 1:** Demographic and baseline characteristics.

	Pooled (n = 118)	VP Tertile		*p*-Value *
		VP < 85% (n = 73)	VP ≥ 85% (n = 45)	
Age, years	77.9 ± 6.7	77.7 ± 6.5	78.4 ± 7.1	0.617
Gender, female, n (%)	60 (50.8)	32 (43.8)	28 (62.2)	0.060
BMI, kg/m^2^	26.8 ± 4.9	26.4 ± 4.7	27.5 ± 5.2	0.218
Previous CAD, n (%)	59 (50.0)	40 (54.8)	19 (42.2)	0.255
Previous CABG, n (%)	20 (16.9)	16 (21.9)	4 (8.9)	0.080
Diabetes mellitus, n (%)	20 (16.9)	29 (39.7)	13 (28.9)	0.322
Hypertension, n (%)	86 (72.9)	53 (72.6)	33 (73.3)	1.000
Dyslipidemia, n (%)	25 (21.2)	18 (24.7)	7 (15.6)	0.354
Prior neurological events, n (%)	8 (6.8)	6 (8.2)	2 (4.4)	0.709
Smoking, n (%)	32 (27.1)	19 (26.0)	13 (28.9)	0.832
Chronic lung disease, n (%)	27 (22.9)	17 (23.3)	10 (22.2)	1.000
Chronic kidney disease, n (%)	31 (26.3)	24 (32.9)	7 (15.6)	0.052
Heart failure, n (%)	24 (20.3)	11 (15.1)	13 (28.9)	0.070
**Laboratory Parameters**				
Baseline creatinine, mg/dL	0.9 (0.8–1.1)	1.0 (0.8–1.2)	0.8 (0.7–1.0)	0.016
Baseline urea, mg/dL	50.3 ± 27.0	53.5 ± 29.7	45.4 ± 21.5	0.115
e-GFR ^†^, mL/min/1.73 m^2^	63.9 ± 20.5	60.4 ± 19.8	69.6 ± 20.6	0.017
Triglyceride, mg/dL	153 ± 71	152 ± 76	156 ± 65	0.731
LDL-C, mg/dL	121.2 ± 36.2	126.0 ±36.0	113.5 ± 35.6	0.069
Total cholesterol, mg/dL	183.9 ± 43.1	189.8 ± 44.8	182.3 ± 41.9	0.369
Glucose, mg/dL	143.0 ± 60.6	148.1 ± 67.2	118.6 ± 33.5	0.007
NT-proBNP, pg/mL	1125 (288–3679)	760 (201–2904)	1682 (670–3769)	0.036
WBC count, ×10^3^/µL	8.2 ± 2.6	8.49 ±2.57	7.76 ±2.78	0.151
Hemoglobin, g/dL	11.4 ± 1.6	11.3 ± 1.7	11.4 ± 1.6	0.733
Platelet count, ×10^3^/µL	235 ± 78	245 ± 88	222 ± 59	0.128
**Echocardiographic Parameters**				
LVEF, %	53.2 ± 7.6	49.4 ± 6.7	59.4 ± 4.2	<0.001
Mean aortic gradient, mm Hg	46.5 ± 11.2	46.0 ± 11.7	47.4 ± 10.7	0.510
Peak aortic gradient, mm Hg	73.7 ± 16.9	73.8 ± 17.2	73.6 ± 16.7	0.950
Aortic valve area, cm^2^	0.69 ± 0.14	0.68 ±0.14	0.72 ±0.15	0.166
Moderate to severe AR, n (%)	28(23.7)	16 (21.9)	12 (26.7)	0.556
Moderate to severe MR, n (%)	30 (25.4)	22 (30.1)	8 (17.8)	0.134
e-SPAP on TR ^‖^, mm Hg	32 (27–40)	34 (28–45)	32 (25–35)	0.045
**Electrocardiographic Characteristics**				
Average PR duration, ms	169 ± 31	168 ± 31	170 ± 34	0.794
Average QRS duration, ms	113 ± 28	114 ± 31	110 ± 23	0.488
Sinus rhythm, n (%)	97 (82.2)	57 (78.1)	91 (82.2)	0.136
Paroxysmal or permanent AF, n (%)	20 (16.9)	15 (20.5)	5 (11.1)	0.184
Low grade AVB, n (%)	14 (11.9)	9 (12.3)	5 (11.1)	0.843
LAFB or LBBB, n (%)	30 (25.4)	19 (26.0)	11 (24.4)	0.848
RBBB, n (%)	8 (6.8)	6 (8.2)	2 (4.4)	0.678

Data are shown in n (%), median (interquartile range; 25th-75th percentiles), and mean ± SD. ^†^ Calculated according to the Chronic Kidney Disease Epidemiology Collaboration (CKD-EPI) equation. ^‖^ Calculated on tricuspid regurgitation peak velocity and right atrial pressure. * A *p*-value of <0.05 was considered statistically significant. Abbreviations: AF: atrial fibrillation; AR: aortic regurgitation; AV: atrioventricular; AVB: atrioventricular block; BMI: body-mass index; BNP: brain natriuretic peptide; CABG: coronary artery bypass surgery; CAD: coronary artery disease; e-GFR: estimated glomerular filtration rate; e-SPAP: estimated systolic pulmonary artery pressure; LAFB: left anterior fascicular block; LBBB: left bundle branch block; LDL-C: low-density lipoprotein cholesterol; LVEF: left ventricular ejection fraction; MR: mitral regurgitation; PPM: permeant pacemaker; RBBB: right bundle branch block; TR: tricuspid regurgitation; VP: ventricular pacing; WBC: white blood cell.

**Table 2:** Procedural, Electrocardiographic and Pacemaker Characteristics.

		Pooled (n = 118)	VP Tertile		*p*-Value *
			VP < 85% (n = 73)	VP ≥ 85% (n = 45)	
Type of THV implanted, n (%)					0.935
	Balloon expandable	102 (86.4)	63 (86.3)	39 (86.7)	
	Self-expandable	16 (13.6)	10 (13.7)	6 (13.3)	
Balloon pre-dilatation, n (%)		79 (66.9)	48 (65.8)	31 (68.9)	0.725
Balloon post-dilatation, n (%)		43 (36.4)	23 (31.5)	20 (44.4)	0.156
Type of PPM implanted, n (%)					0.573
	Single chamber	54 (45.8)	35 (47.9)	19 (42.2)	
	Dual chamber	64 (54.2)	38 (52.1)	26 (57.8)	
Post-TAVR temporary pacing, n (%)		64 (54.2)	30 (41.1)	34 (75.6)	<0.001
Time to PPM implantation, day		4.8 ± 2.9	6.2 ± 2.6	2.6 ± 1.3	<0.001
Reason for PPM implantation, n (%)					<0.001
	Complete high degree AVB	47 (39.8)	16 (21.9)	32 (71.1)	
	Paroxysmal high degree AVB	28 (23.7)	23 (31.5)	5 (11.1)	
	New onset BBB	16 (13.6)	16 (21.9)	2 (4.4)	
	Symptomatic bradycardia	18 (15.3)	10 (13.7)	6 (13.3)	
	Other reason	9 (7.6)	9 (12.3)	0 (0.0)	
All PPM complications, n (%)		3 (2.5)	2 (2.7)	1 (2.2)	0.886
**Echocardiography, ECG and Pacemaker Features**					
LVEF, %		54.2 ± 6.6	50.6 ± 6.6	54.2 ± 5.8	0.003
LVEF change, %		0 (−5–0)	0 (−1–5)	−5 (−8–0)	<0.001
Mean TV gradient, mm Hg		10.7 ± 4.5	10.6 ± 3.8	10.9 ± 5.5	0.715
Peak TV gradient, mm Hg		18.4 ± 7.3	17.9 ± 5.3	19.3 ± 9.6	0.304
e-SPAP on TR ^†^, mm Hg		35.5 ± 8.9	29.7 ± 9.1	35.5 ± 8.9	0.001
New onset AF, n (%)		9 (7.6)	9 (12.3)	0 (0.0)	0.014
RBBB with LAFB/LPFB, n (%)		12 (10.2)	10 (13.7)	2 (4.4)	0.106
Average PR duration ^‖^, ms		187.0 ± 43.5	185.2 ± 41.8	196.1 ± 52.9	0.431
Average QRS duration ^‖^, ms		136.2 ± 23.3	135.3 ± 24.6	141.6 ± 12.9	0.411
Percentage of right VP, %		61.6 ± 34.5	39.7 ± 26.0	97.2 ± 4.3	<0.001
	Right VP ≥ 90%, n (%)	44 (37.3)	0 (0.0)	44 (97.8)	<0.001
	Right VP ≥ 95%, n (%)	33 (28.0)	0 (0.0)	33 (73.0)	<0.001
Absence of intrinsic heart activity ^§^, n (%)		26 (22.0)	0 (0.0)	26 (57.8)	<0.001
HF-caused hospitalization, n (%)		25 (21.2)	13 (17.8)	12 (26.7)	0.253
1-year all-cause mortality ^¶^, n (%)		22 (18.6)	11 (15.1)	11 (24.4)	0.204

Data are shown in n (%), median (interquartile range; 25th–75th percentiles), and mean ± SD. ^†^ Calculated on TR peak velocity and right atrial pressure. ^‖^ Patients with complete high degree AVB were excluded. ^§^ Only percentage of right VP = 100%. ^¶^ 19/22 were attributable to cardiac causes and 3 to non-cardiac causes. * A *p*-value of <0.05 was considered statistically significant. Abbreviations: AF: atrial fibrillation; AV: atrioventricular; AVB: atrioventricular block; BBB: bundle branch block; LAFB: left anterior fascicular block; e-SPAP: estimated systolic pulmonary artery pressure; HF: Heart failure; LBBB: Left bundle branch block; LPFB: left posterior fascicular block; LVEF: left ventricular ejection fraction; PPM: permeant pacemaker; RBBB: right bundle branch block; TAVR: transcatheter aortic valve replacement THV: transcatheter heart valve TR: tricuspid regurgitation; VP: ventricular pacing.

**Table 3:** One-year mortality and first heart failure hospitalization according to VP rate after TAVR in patients with permanent pacemaker implantation.

VP Rate		Population		Multivariable Cox Regression		
		Deceased (n = 22)	Alive (n = 96)			
				a-HR ^†^	95% CI	*p*-Value *
Continuous, %		70.5 ± 33.1	59.5 ± 35.0	1.02	0.99–1.04	0.077
Categorized, n (%)						
	<85%	11 (15.1)	62 (84.9)	(1) ref		
	≥85%	11 (24.4)	34 (75.6)	2.04	0.61–6.78	0.244

Data is shown in n (%), mean ± standard deviation. ^†^ Note that the cohort was adjusted for the following set of covariates: age, gender, NT-proBNP levels, e-SPAP, and LVEF. * A *p*-value of <0.05 was considered statistically significant. Abbreviations: a-HR: Adjusted hazard ratio; CI: confidence interval; VP: ventricular pacing.

## Data Availability

Data are available on request due to privacy and ethical restrictions.

## Supplementary Materials

The following supporting information can be downloaded at: https://www.mdpi.com/article/10.3390/medicina61101758/s1, Figure S1: X-tile analysis demonstrating the optimal cut-off point of VP, with corresponding χ^2^ statistics and *p*-values used to define risk stratification groups.
